# Tibiofemoral rotation is significantly higher in patients with patellofemoral maltracking and torsional deformity

**DOI:** 10.1007/s00402-026-06198-3

**Published:** 2026-02-02

**Authors:** Sina Gräber, Felix Hüttner, Andrzej Jasina, Parisa Pourostad, Turgay Efe, Thomas Tischer, Jörg Harrer, Christoph Lutter, Felix Ferner

**Affiliations:** 1 Department of Orthopaedic Surgery, Rostock University Medical Center, Rostock, Germany; 2 Department of Orthopaedic Surgery, Sanaklinikum Lichtenfels, Lichtenfels, Germany; 3 Orthopaedicum Lich, Lich, Germany; 4 Department of Orthopaedic Surgery, Malteser Waldkrankenhaus Erlangen, Erlangen, Germany

**Keywords:** Knee, Patellofemoral maltracking, Patellofemoral instability, Tibiofemoral rotation, Knee version, Torsional deformity

## Abstract

**Introduction:**

Tibiofemoral (TF) rotation, the relative rotational alignment between femur and tibia in the axial plane at the level of the knee has been investigated as a parameter of joint alignment. Its influence on patellofemoral pathologies is largely unknown. Within this cross sectional study, it was hypothesized that TF rotation is increased in patients with symptomatic torsional femoral or tibial deformity and associated patellofemoral maltracking compared to healthy individuals.

**Methods:**

This single-center-study included patients with patellofemoral maltracking, including patellofemoral instability, who underwent tibial and/or femoral derotational osteotomy (surgery 2019–2024) and for whom preoperative torsional MRI was available. Torsion was measured according to Waidelich et al. and TF rotation defined as the angle between a tangent on the dorsal femoral condyles and a tangent on the dorsal tibial plateau. Positive values indicating external, negative values internal rotation. Tibial-tuberosity-trochlea-groove (TT-TG) distance was measured additionally. Interrater reliability was calculated between measurements of orthopaedic surgeons and musculoskeletal radiologists. Results were compared with those of a healthy control group that had previously been published.

**Results:**

86 cases were included (age 25.0 ± 9.0, 78% females). Level of derotational osteotomy was femoral in 36% (*n* = 31), tibial in 26% (*n* = 22), double-level in 38% (*n* = 33). Interrater reliability was good to excellent for all parameters measured. TF rotation (5.6 ± 6.7°) was significantly increased (*P* < .001) compared to healthy subjects (1.3 ± 3.9°). 50% of the cases suffered from patellofemoral instability. Among these, TF rotation was significantly increased compared to cases without instability (7.5 ± 7.3° vs. 3.8 ± 5.5°; *P* = .011). TF rotation correlated moderately negative with tibial torsion (r_s_=-0.284; *P* = .008) and moderately positively with TT-TG-distance (r_s_=0.487, *P* < .001). There was no significant difference in TF rotation between different osteotomy levels.

**Conclusion:**

TF rotation is significantly increased in patients with symptomatic torsional deformity and associated patellofemoral maltracking and should therefore be included in future investigations for establishing further treatment recommendations.

## Introduction

 Patellofemoral maltracking, including patellofemoral instability is often caused by osseous factors such as patella alta, dysplasia of the trochlea, femorotibial valgus deformity or femoral or tibial torsional deformity. In most cases a combination of different deformities with different severity is common and operative treatment has to be considered in many cases [1–6]. Next to medial patellofemoral ligament (MPFL) reconstruction, in severe cases corrections of osseous factors are incremental parts of surgical strategies. Hereby trochleoplasty, distalization/medialization of the tibial tubercle, varisation osteotomy or derotational osteotomy might be necessary [6–14]. Nevertheless, some of these patients suffer persistent pain or instability after surgical procedures, even if each known predisposing factor for a patellofemoral malalignment was surgically addressed correctly according to the existing knowledge. The literature reports redislocation rates of between 1% and 7% following MPFL reconstruction (combined with or without trochleoplasty or tibial tuberosity osteotomy) [15–19]. Although the redislocation rate after derotational femoral osteotomy is lower [20, 21], a J-sign persists in 28% of cases [21]. In addition, anterior knee pain persists in 7.6% of cases after derotational osteotomy [20]. This shows that the biomechanic of the patellofemoral joint is not yet fully understood [22, 23]. In this context the tibiofemoral (TF) rotation or “knee version” as it is originally described has been investigated, which is the intraarticular rotation of the knee and defined as the angle between the tangent of the dorsal femoral condyles and the tangent of the dorsal tibial plateau [24]. TF rotation has been rarely discussed in the literature but recently gained more attention as Flury et al. found the winking sign as an indicator for an increased tibiofemoral rotation in anterior-posterior radiograph [25]. The normal values for TF rotation in a healthy population have already been determined [26]. In addition, previous studies revealed an increased TF rotation in adolescents, patients with an increased femoral torsion and cases of patellofemoral instability [27–29]. To the best of our knowledge, there is no study in the existing literature that investigated the TF rotation in a large group of patients presenting with patellofemoral maltracking and a torsional deformity (including single tibial torsional deformity) with a standardized torsional MRI protocol.

It was therefore hypothesized that TF rotation would be increased in patients with torsional deformities of the tibia, the femur or both and patellofemoral maltracking. Therefore, TF rotation was measured in patients with a torsional deformity and compared to normal values.

## Methods

In this single-center study patients with patellofemoral maltracking, including patellofemoral instability and existing torsional deformity were included. The ethics committee of the University Medical Centre Rostock gave ethical approval (A 2020-0273). Inclusion criteria were surgically corrected femoral and/or tibial torsion between 2019 and 2024 in patients with closed physis of the distal femur and proximal tibia (evaluated in MRI) and a preoperative Magnetic resonance imaging (MRI) to assess torsional deformities of the lower limb. Patients who had previously undergone MPFL reconstruction or bony correction on the same knee were excluded. MRI examinations were all performed according to the same protocol, which was published by Huettner et al. 2023 [26] with a 1.5-T scanner (Magnetom Aera; Siemens). For all patients included, the preoperative values of femoral and tibial torsion, TF rotation and tibial-tuberosity-trochlea-groove (TT-TG) distance were retrospectively analyzed by two trained orthopaedic surgeons (SG, FH) using a commercial picture archiving and communication system (DeepUnity Diagnost Version 2.0.2.2; Daedalus DACH). Measurements from an experienced musculoskeletal radiologist were used for calculating interobserver reliability for absolute agreement by determining the intraclass correlation coefficient (ICC).

**Fig. 1:**
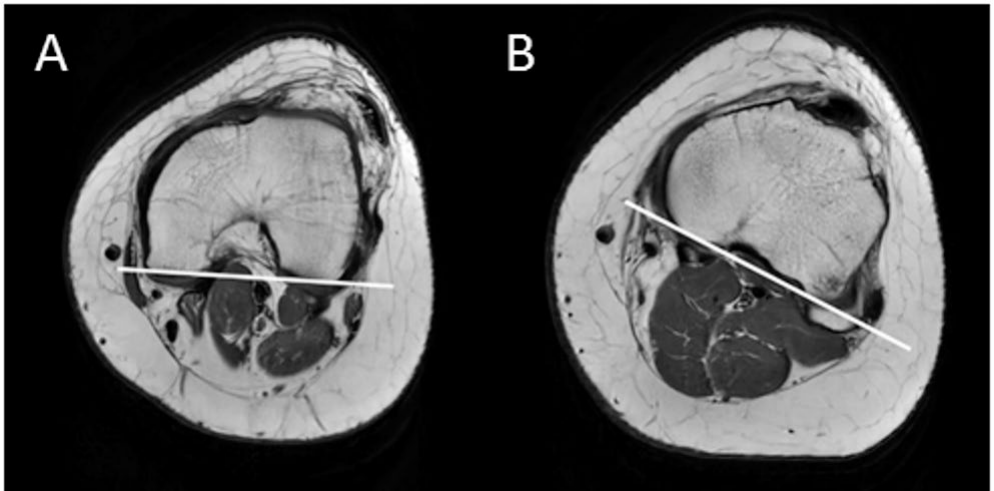
Torsional MRI of a left knee. Drawn lines showing the dorsal femoral condyle tangent line (**A**) and the dorsal tibial head tangent line (**B**). The angle between the two tangents is the TF rotation

The torsion measurements were performed according to Waidelich et al. [30]. As previously described by Eckhoff et al. TF rotation was defined as the angle between a tangent to the dorsal femoral condyles and a tangent to the dorsal tibial plateau (Fig. 1) [24]. Additionally TT-TG distance was measured [31]. For all angles measured, positive values indicate an external torsion, negative values an internal torsion.

The results were compared to those of a control group, containing 100 healthy individuals with no history of patellofemoral maltracking, torsional deformity or any other history of lower extremity injuries. This is the same cohort used by Huettner et al. to define standard values for TF rotation [26]. The original data from this study were available.

### Statistical evaluation

For statistical evaluation IBM^®^ SPSS^®^ Statistics (v27.0.1; IBM Deutschland GmbH, Ehningen, Germany) was used. Comparisons were made between groups considering different pathologies (f. e. patellofemoral maltracking with and without patellofemoral instability or different osteotomy levels) or different sexes. Hereby, all patients were distributed either to the “maltracking group” if they had no history of patella dislocation and if they had one or recurrent patella dislocations they were distributed to the “instability group”. Cases in which the TF rotation deviated by 2 standard deviations or more from the normal value for the healthy population were classified as highly pathological.

The data was tested for normal distribution using Shapiro-Wilk test. Depending on this, pairwise comparisons were performed using t-Test or Mann-Whitney-U-Test. ANOVA or ANOVA-on-ranks were used to compare several groups. Depending on the normal distribution, the Pearson´s correlation coefficient or Spearman´s correlation coefficient was calculated. The significance level was set at 5%. Data is presented as mean ± standard deviation (minimum – maximum).

## Results

A total of 82 patients met the inclusion criteria whereby in 4 cases both legs were treated. This results in 86 cases included in the study. For further information see Table 1. Patients had a mean age of 25.0 ± 9.1 (12–53) years. The preoperative MRI showed an average TF rotation of 5.6 ± 6.7 (−6.9–36.5)°. Femoral and tibial torsion as well as TT-TG distance are listed in Table 3. Interobserver reliability, was good to excellent for all measured parameters (Table 2).

**Table 1:** Sex and affected side of the cases included in this study

	Overall cases (n = 86)
Female : Male (n)	67:19
Affected side (n):	
left	32
right	54

### Comparison to healthy individuals

Compared to the previously published values of a healthy population [26], there are highly significant differences to the present study. In patients with patellofemoral disorders, the TF rotation is significantly increased (*P* <.001) with an average of 5.6 ± 6.7 (−6.9–36.5)° external rotation compared to healthy subjects, who had a mean TF rotation of 1.3 ± 3.9 (−8.7–11.7)° [26]. In 22% (*n* = 19) of patients with patellofemoral disorder, there was a highly pathological TF rotation. Femoral internal and tibial external torsion as well as TT-TG distance were highly significantly increased in patients with patellofemoral disease compared to the healthy control group. Details are shown in Table 3.

**Table 2:** Intraclass correlation coefficient for determining the interobserver reliability. The 95% confidence interval is given in brackets

	Interobserver reliability
Femoral torsion	0.895 [0.835–0.934]
Tibial torsion	0.987 [0.979–0,992]
TF rotation	0.988 [0.979–0.994]
TT-TG	0.934 [0.891–0.960]

**Table 3:** Comparison of femoral and tibial torsion, TF rotation and TT-TG distance between patients with patellofemoral disorder (present study) and healthy individuals (original data from the study by Huettner et al.) [26]

		Patellofemoral disorder	Healthy population [26]	*P*
Number	86		200	
Age (years)	25.0 ± 9.1 (12 to 53)		26.5 ± 5.8 (18 to 40)	0.082
Femoral torsion (°)	−36.2 ± 10.1 (−67.2 to −5.6)		−23.8 ± 9.7 (−46.2 to 1.6)	< 0.001
Tibial torsion (°)	43.7 ± 9.8 (1.1 to 65.7)		33.2 ± 7.4 (16.4 to 50.3)	< 0.001
TF rotation (°)	5.6 ± 6.7 (−6.9 to 36.5)		1.3 ± 3.9 (−8.7 to 11.7)	< 0.001
TT-TG (mm)	18.9 ± 4.5 (9.4 to 34.6)		13.4 ± 3.7 (5.3 to 23.5)	< 0.001

Negative values indicating internal torsion, positive values external torsion

### Comparison between patellofemoral maltracking with and without patellofemoral instability

In the present study, 50% (*n* = 43) of the cases showed patellofemoral instability and 50% (*n* = 43) not. Among patients with patellofemoral instability, TF rotation with an average of 7.5 ± 7.3 (−5.7–36.5)° was significantly increased compared to those with patellofemoral maltracking without instability (3.8 ± 5.5 (−6.9–17.0)°; *P* =.011). As well, the proportion of highly pathological TF rotation values was significantly higher in the instability group (30%, *n* = 13) than in the maltracking group (14%, *n* = 6). TT-TG distance was higher in the patellofemoral instability group (*P* <.001), whereas tibial external torsion was on average higher among patients without patellofemoral instability (*P* =.009). No significant difference was found concerning femoral torsion (Table 4).

**Table 4:** Comparison of femoral and tibial torsion, TF rotation and TT-TG distance between patients with patellofemoral maltracking with instability and without instability

Patellofemoral maltracking:	With instability	Without instability	*P*
Number	43	43	
Femoral torsion (°)	−34.4 ± 9.4 (−52.0 to −5.6)	−38.1 ± 10.6 (−67.2 to −18.9)	0.089
Tibial torsion (°)	41.0 ± 7.6 (24.8 to 59.6)	46.4 ± 11.1 (1.1 to 65.7)	0.009
TF rotation (°)	7.5 ± 7.3 (−5.7 to 36.5)	3.8 ± 5.5 (−6.9 to 17.0)	0.011
TT-TG (mm)	20.7 ± 4.7 (11.8 to 34.6)	17.1 ± 3.6 (9.4 to 24.5)	< 0.001

Negative values indicating internal torsion, positive values external torsion

### Relationship between TF rotation and torsional deformity

The preoperative MRI showed no difference in TF rotation depending on osteotomy-level. The same applies to the TT-TG distance. Details are shown in Table 5. The proportion of highly pathological TF rotation values was 26% (*n* = 8) for patients with femoral torsional deformity, 18% (*n* = 4) for patients with tibial torsional deformity and 21% (*n* = 7) for patients with a combined torsional deformity.

In the overall cases there was a moderate negative correlation between TF rotation and tibial torsion (r_s_ = −0.284; *P* =.008) and a moderate positive correlation between TF rotation and TT-TG distance (r_s_ = 0.487; *P* <.001). TF rotation and femoral torsion were not significantly correlated (Table 6).

**Table 5:** Comparison of femoral and tibial torsion, TF rotation and TT-TG distance between different osteotomy-levels in the preoperative MRI

	Femoral dOT	Tibial dOT	Tibial & Femoral dOT	*P*	
Number	31	22	33		
Femoral torsion (°)	−41.5 ± 8.5 (−67.2 to −28.6)	−26.7 ± 5.2 (−34.9 to −18.9)	−37.6 ± 9.8 (−55.0 to −5.6)	< 0.001	
Tibial torsion (°)	35.7 ± 9.4 (1.1 to 53.6)	47.5 ± 7.5 (33.3 to 60.2)	48.7 ± 6.4 (40.0 to 65.7)	< 0.001	
TF rotation (°)	6.5 ± 5.2 (−5.5 to 17.7)	5.1 ± 6.3 (−6.9 to 20.2)	5.2 ± 8.3 (−5.7 to 36.5)	0.457	
TT-TG (mm)	19.7 ± 5.0 (10.2 to 34.6)	18.8 ± 4.4 (13.2 to 30.2)	18.1 ± 4.1 (9.4 to 25.6)	0.221	

dOT refers to derotational osteotomy. Negative values indicating internal torsion, positive values external torsion

*P* value refers to the comparison between all three groups

### Comparison between male and female

Females (*n* = 67) were more frequently represented in this study than males (*n* = 19). Even if the TF rotation among males was lower (3.7 ± 6.7 (−6.0–17.0)°) compared to females (6.2 ± 6.7 (−6.9–36.5)°), there was no statistically significant difference between them and the proportion of highly pathological TF rotation value was similar (21%/*n* = 4 among males and 22%/*n* = 15 among females). Further information is provided in Table 7.

**Table 6:** Correlation between TF rotation and the other parameters measured (femoral torsion, tibial torsion, TT-TG distance)

	TF rotation	
	r_s_	P
Femoral torsion	−0.159	0.144
Tibial torsion	−0.284	0.008
TT-TG	0.487	<0.001

r_s_ = Spearman`s correlation coefficient

**Table 7:** Comparison of femoral and tibial torsion TF rotation and TT-TG distance between sexes

	Male		Female	*P*
Number	19		67	
Femoral torsion (°)	−33.1 ± 9.6 (−52.8 to −18.9)		−37.1 ± 10.2 (−67.2 to −5.6)	0.038
Tibial torsion (°)	51.8 ± 7.1 (40.0 to 65.7)		41.4 ± 9.3 (1.1 to 61.2)	0.001
TF rotation (°)	3.7 ± 6.7 (−6.0 to 17.0)		6.2 ± 6.7 (−6.9 to 36.5)	1.000
TT-TG (mm)	18.7 ± 4.2 (12.6 to 26.8)		18.9 ± 4.6 (9.4 to 34.6)	0.909

Negative values indicating internal torsion, positive values external torsion

## Discussion

The most important finding of the study is that TF rotation is significantly increased in patients with patellofemoral maltracking not only in cases of femoral torsional deformity, but also in cases of isolated tibial torsional deformity compared to a healthy population. Hereby, patients with patellofemoral instability present with an even more increased TF rotation than patients with solely patellofemoral maltracking without instability. However, TF rotation in the overall cohort with patellofemoral maltracking correlates significantly negatively with tibial torsion. These findings strongly suggest measuring TF rotation in all patients with patellofemoral maltracking and a torsional deformity.

Since normal values for TF rotation have been determined, this is to the best of our knowledge the first study to focus exclusively on torsional deformities and the first to include isolated torsional deformities of the tibia, showing that TF rotation is significantly higher in these patients compared to healthy controls. There are a few studies that have analyzed TF rotation in patients with patellofemoral instability with and without concomitant torsional deformities in the past and measured average values between 6.9° and 8.8° [27, 28, 32]. This is slightly higher compared to the 5.6 ± 6.7° measured in the present study. One reason might be that they included patients with different pathologies, not only torsional deformities in their studies or due to the fact that within the studies by Lin et al. and Bernholt et al. the measurements were performed on standard MRIs of the knee joint [27, 32]. Standard MRIs are usually performed under less controlled conditions than a torsional MRI. For example, different degrees of flexion may be present, and the ankle joint is not fixed, which could influence the TF rotation if the knee is even slightly flexed. Recently published findings suggest that the measurement of TF rotation is not consistent and not reliable in different degrees of flexion [33]. It should be noted, however, that in the study by Lin et al. the standard MRIs were performed with an extended knee joint [32] and therefore the limitations mentioned above have to be considered carefully.

Huettner et al. [26] were able to demonstrate a significant difference in TF rotation between men and women in their study, with women showing an elevated TF rotation of 2.4°. In the present study, women showed an average 2.5° higher TF rotation than men. However, this difference was not statistically significant. This is probably due to a relative gender mismatch with only 19 included males. Therefore, future investigations with a higher number of cases must reevaluate if there is a significant difference in TF rotation in patients with a patellofemoral maltracking between males and females.

Wu et al. [34] analyzed tibiofemoral rotation in 118 patients with recurrent patella dislocation in a retrospective analysis and found a mean value for TF rotation of 12 *±* 6°. The authors concluded that the severe cases of patellofemoral maltracking are associated with an increased TF rotation. Nevertheless, the measurement method in this study consisted of reconstructing a 3D-model from the hip-knee-ankle CT-scan according to the method of Takagi et al. [35]. This differs to the so far established method on torsional MRI, where the normal values for TF rotation are well known. Therefore, the measurement of TF rotation can only be performed reliable with the knee in full extension with a standardized protocol as it is available for torsional MRI or CT scans [26]. There are studies that deal with other possible measurement methods. For example, Jud et al. 2024 described the tibial tuberosity torsion measured in CT scans as the most predictable value in patients with patellofemoral instability [28]. Whether this value offers an advantage over a separate measurement of TT-TG distance and TF rotation in clinical practice remains a question for further studies.

Currently, most surgeons aim for the normal value of femoral or tibial torsion as the goal of torsional corrective osteotomy, which was also performed in the current study. After derotational osteotomies some patients still suffer persistent patellofemoral pain or instability. Liße et al. [36] reported a persistent patellofemoral instability in 3 of 9 cases after double-level derotational osteotomy, whereas Figueiredo et al. [20] reported in a recent review of 564 knee derotational osteotomies about 7.5% of the patients with persistent anterior knee pain postoperative. The results of the current study strongly suggest measuring the TF rotation prior to derotational osteotomy and including the TF rotation into the diagnostical algorithm in patients with a torsional pathology. Moreover, it may be considered to involve a (pathological increased) TF rotation into preoperative planning. This would possibly result in adjusting the correction angle of the torsional correction by the amount of TF rotation. It should be noticed that this is not scientifically proven yet and future investigations are necessary to confirm this theoretical consideration.

A significantly increased TF rotation in patients with patellofemoral instability compared to patients with a maltracking was found within this study, which indicates that the cases with instability are the more severe cases or vice versa instability may be able to increase the TF rotation itself over time. Jud et al. [29] investigated the change of TF rotation after derotational osteotomy. They found no significant decrease of tibiofemoral rotation in 40 femoral derotational osteotomies whereas the preoperative mean value in their cohort was 9.9 ± 6.2°, which is slightly higher than in the femoral cohort (6.5 ± 5.2 °) from the present study. There is no information in the current literature about the possible change of TF rotation after surgery which refer exclusively to tibial or double-level osteotomies. Even though Jud et al. included 16 cases of double-level osteotomies in the study mentioned above [29]. Currently, there is only one study that found a correction of TF rotation by an operative procedure: Hartmann et al. documented a decrease in TF rotation after tibial tubercle osteotomy [37].

A surgical solution cannot be directly deviated from the findings of the present study, which was not the goal of this investigation. But the measurement of the TF rotation should be included in the standard procedure when patients with a patellofemoral problem are undergoing torsional investigations. Hereby, the first step is to diagnose the critical cases with an increased TF rotation, the second step is to find surgical solutions for these cases, which is a task for the near future.

The findings of the current study strongly suggest implementing the measurement of TF rotation on a standardized torsional MRI protocol into the diagnostical algorithm in patients with a patellofemoral malalignment.

## Limitations

The measurement of the TF rotation is performed in full extension. It is not clear how TF rotation changes during knee flexion. Some severe cases of patellofemoral instability suffer instability, especially in deep flexion which still is an unsolved problem, and it is not clear if these cases can be solved with the current concept. Further studies must be obtained investigating the TF rotation in different degrees of knee flexion. Furthermore, this study does not take into account other risk factors for patellofemoral instability, such as patella alta or dysplasia of the trochlea. Nevertheless, the measurements obtained in this study were all performed according to the same MRI protocol and according to the exact same protocol as the measurements of the “normal values” by Huettner et al. [26] were obtained. Whereas in Huettner´s cohort only adults (*≥* 18 years) were included, in the current study also adolescents were recorded – however, following the inclusion criteria “closed physis” all included cases were considered skeletal mature. The total number of 86 cases for the measurement of TF rotation seems to be sufficient, whereas the number of 43 cases for patients with a patellofemoral instability and maltracking is relatively low. Further measurements, especially for the different subgroups (e.g. females/males) with a higher number of cases should be performed in the future to confirm the findings of the current study.

## Conclusion

TF rotation is significantly increased in patients with symptomatic torsional deformity and associated patellofemoral maltracking compared to healthy individuals. In patients with patellofemoral instability, the TF rotation is even higher compared to those with patellofemoral maltracking without instability. These results make it likely that TF rotation is an important parameter in pathologies of the patellofemoral joint and should be measured as such. Future studies are needed to determine the impact of an increased TF rotation on treatment recommendations in patients with patellofemoral pathologies and influence on surgical goal of correction during torsional osteotomies.

## Data Availability

The data that support the findings of this study are not openly available due to reasons of sensitivity and are available from the corresponding author upon reasonable request.
